# Release rate and antimicrobial activity of gentamicin salts as coating for bone allografts

**DOI:** 10.1186/1753-6561-5-S6-P200

**Published:** 2011-06-29

**Authors:** DC Coraca-Huber, K-D Kühn, M Fille, J Hausdorfer, L Fuderer, M Nogler

**Affiliations:** 1 Experimental Orthopedics, Medical University Innsbruck - Austria, Innsbruck, Austria; 2 Pedriatic and Adolescence Surgery, Medical University of Graz, Graz, Austria; 3 Hygiene and Medical Microbiology, Medical University Innsbruck, Innsbruck, Austria; 4 Experimental Orthopedics, Medical University Innsbruck, Innsbruck, Austria

## Introduction

The rapidly increasing number of joint arthroplasties performed around the world has seen a rising number of complications. Since the contamination is normally associated to metal surfaces and dead tissues like bone grafts, local delivery of antibiotics is an option for therapy and prophylaxis of implant associated infections.

## Objectives

In this study, we tested the delivery rate and antibiotic activity of gentamicin palmitate mixed with gentamicin sulfate as coating for bone allografts.

## Methods

Bone chips were obtained by morsellising femur heads and impregnated with GS+GP, GS pure and Herafill^®^ as control. The samples were analysed before and after 1 month of storage at -80°C. The drug release rate was evaluated *in vitro* after 0, 1, 4, 8 and 12 hours and 1, 2, 3, 4, 5, 6 and 7 days. Antimicrobial efficacy was determined against *S. aureusand S. epidermidis*.

## Results

The released rate of GS pure and GS+GP was similar along time and significant lower than Herafill^®^. However, for both strains, GS+GP and GP pure were more effective than Herafill^®^. *S. epidermidis* is significantly more susceptible to GS+GP, GP pure and Herafill^®^ than *S. aureus*. No significant differences were observed before and after the storage of samples.

## Conclusions

The capacity of bone grafts to act as antibiotic carrier has been confirmed in this study. The lower delivery rate of GS+GP compared to GS pure and Herafill® can be an advantage for longer release time increasing the local protection against infections. Short-therm storage at -80°C does not compromise the coating activity.

## Disclosure of interest

None declared.

